# Limited intercarpal fusion versus proximal row carpectomy in the treatment of SLAC or SNAC wrist, results after 3.5 years

**DOI:** 10.1186/s13018-023-04177-7

**Published:** 2023-09-13

**Authors:** Robert Gvozdenovic, Martina Agerskov Schioedt, Lars Solgaard, Lars Soelberg Vadstrup, Niels Henrik Soee

**Affiliations:** 1 Department of Hand Surgery, Herlev/Gentofte University Hospital of Copenhagen, Hospitalsvej 1, 2900 Hellerup, Denmark; 2 Faculty of Health and Medical Sciences, Institute of Clinical Medicine, University of Copenhagen, Blegdamsvej 3B, 2200 Copenhagen N, Denmark

**Keywords:** SLAC, SNAC, Proximal row carpectomy, Limited intercarpal arthrodesis, Midcarpal fusion

## Abstract

The present study compares the postoperative clinical, radiological, and patient-reported functional results between the surgical procedures Proximal Row Carpectomy and Limited Carpal Fusion, in the treatment of SLAC and SNAC conditions of the wrist. 15 Proximal Row Carpectomy patients and 45 Limited Carpal Fusion patients were included in the study. Postoperative outcomes were assessed and compared for pain at load, range of motion, grip strength, Quick-DASH, and satisfaction. A radiological assessment was performed at the follow-up. The Proximal Row Carpectomy patients had a mean age of 60 years (range 31–77) and a mean follow-up of 42 months. The Limited Carpal Fusion patients had a mean age of 58 years (range 35–76) and a mean follow-up of 41 months. The patients treated with Limited Carpal Fusion performed significantly better regarding pain, radial-ulnar motion, and the Quick-DASH (*p* = 0.002, *p* = 0.003, and *p* = 0.002), respectively. The grip strength difference between the treatment groups was stratified for gender and was found significantly better for men in the LCF-treated patients, but not different for women (*p* = 0.03, *p* = 0.26), respectively. Differences in flexion–extension between the groups were insignificant (*p* = 0.525). A higher conversion rate to total wrist fusion was observed in the patients treated with the Proximal Row Carpectomy. All the Proximal Row Carpectomy patients had osteoarthritis at follow-up, whereas it was seen in 19% of the Limited Carpal Fusion patients. The patient-reported satisfaction was substantially better for the Limited Carpal Fusion patients. In conclusion, among patients treated for SNAC and SLAC wrist conditions, besides the findings of flexion–extension, and grip strength which were found without difference for women the findings are in favour of Limited Carpal Fusion compared to Proximal Row Carpectomy. Further, preferably prospective studies are needed to confirm or reject our findings.

*Level of evidence*: Retrospective, comparative cohort study, level III.

## Introduction

Scapholunate advanced collapse (SLAC) and scaphoid nonunion advanced collapse (SNAC) are frequent patterns of degenerative arthritis in the wrist [1]. There are different motion-preserving and pain-reducing salvage procedures for the treatment of SLAC and SNAC, which include Proximal Row Carpectomy (PRC), four-corner fusion (4CF), or limited carpal fusions (LCF) [2, 3]. All the procedures are well established in the treatment of SLAC and SNAC, but there is still no consensus on which technique gives the best outcomes [4, 5]. Traditionally, 4CF and PRC are the most common procedures when choosing a salvage procedure. Recently, limited carpal fusion options have been developed. Limited carpal fusions use less hardware and are less invasive compared to traditional 4CF. By using headless compression screws for proper fixation there is, in comparison to earlier studies on 4CF, a lower nonunion rate in LCF [6].

Numerous studies compare the outcomes of 4CF with PRC [7–11]. Generally, when comparing PRC with 4CF, PRC results in a better range of motion (ROM) and 4CF in better grip strength. Nevertheless, 4CF has more complications due to hardware issues, the development of dorsal impingement, and nonunion in some patients. Patients who have undergone PRC are thought to have a greater risk of developing osteoarthritis than those who have had 4CF. This is probably caused by the different diameters of the articular surfaces between the capitate and radius [12]. Nevertheless, the two procedures are comparable and seem to result in similar outcomes [4, 9]. Comparative studies between PRC and LCF are lacking in the literature. Since the LCF procedures are less invasive [13], there is a knowledge gap if the LCF-treated patients operated on with headless compression screws could perform better than traditional 4CF patients, in comparison with PRC-treated patients.

Thus, this retrospective study aims to compare postoperative clinical, functional, and radiological outcomes among patients treated for the consequences of a SLAC or SNAC condition of the wrist. The null hypothesis is that there is no difference between the treatment methods, Proximal Row Carpectomy or Limited Carpal Fusion.

## Method and material

### Study design and patients

Between August 2014 and February 2021, 16 patients underwent the PRC procedure, and 45 patients were operated on with an LCF technique due to the development of a SLAC or SNAC wrist. The inclusion criteria for this study were: All patients with the age of above 18 years referred consecutively to our institution for surgical treatment of the painful wrist caused by SLAC or SNAC wrist condition. Exclusion criteria were age under 18 years and follow-up of less than 6 months. 15 of the 16 patients treated with the PRC technique were included. One patient from this group died, 6 months after the operative treatment, by causes not related to treatment, and was therefore excluded from the study. All patients treated with the LCF technique (n = 45) were included in this study. All procedures were performed by specialized hand surgeons and the choice of the surgical method was the treating surgeon´s choice. According to Tang and Giddins, there were five different surgeons with a high surgical experience level (three level III, two level IV) in each treatment group [14]. The demographic data of the patients are presented in Table 1.

**Table 1:** Patient demographics

Patients demographics	PRC	LCF
Total number of patients	15	45
Women	10	15
Men	5	30
Age^a^	60 (31–77)	58.4 (35–79)
Follow-up^b^	42 (6–71)	41 (12–68)
Dominant hand	9 (64%)	26 (65%)^c^
Tobacco smokers	3 (20%)	10 (23%)^d^
Alcohol consumers	7 (47%)	31 (69%)
Diabetes	1 (7%)	4 (9%)
Other comorbidities^e^	7 (47%)^f^	17 (41%)^g^

^a^Expressed as mean (range), in years

^b^Expressed as mean (range), in months

^c^Number of patients: 40. Missing data on 5 patients

^d^Number of patients: 44. Missing data on 1 patient

^e^Expressed as number of patients with at least one comorbidity

^f^Arterial hypertension: 4, hypercholesterolaemia: 2, chronic obstructive pulmonary distress syndrome: 1, polyarthritis: 1, osteoporosis: 1, serum negative rheumatoid arthritis: 1, heart failure: 1, colitis ulcerosa: 1

^g^Total number of patients: 41. Missing data on 4 patients. Arterial hypertension: 13, hypercholesterolaemia: 6, chronic obstructive pulmonary distress syndrome: 2, polyarthritis: 1, osteoporosis: 1, hypothyroid: 1

In both groups, there were patients who experienced previous surgery, 8/15 from the PRC group, and 20/45 from the LCF group (*p* = 0.27). Of these, two PRC patients (13%), and five LCF patients (11%) had > 1 surgery performed previously. Besides differences in sex dispersion, both groups were similar.

### Operative techniques and postoperative management

#### PRC

**Fig. 1:**
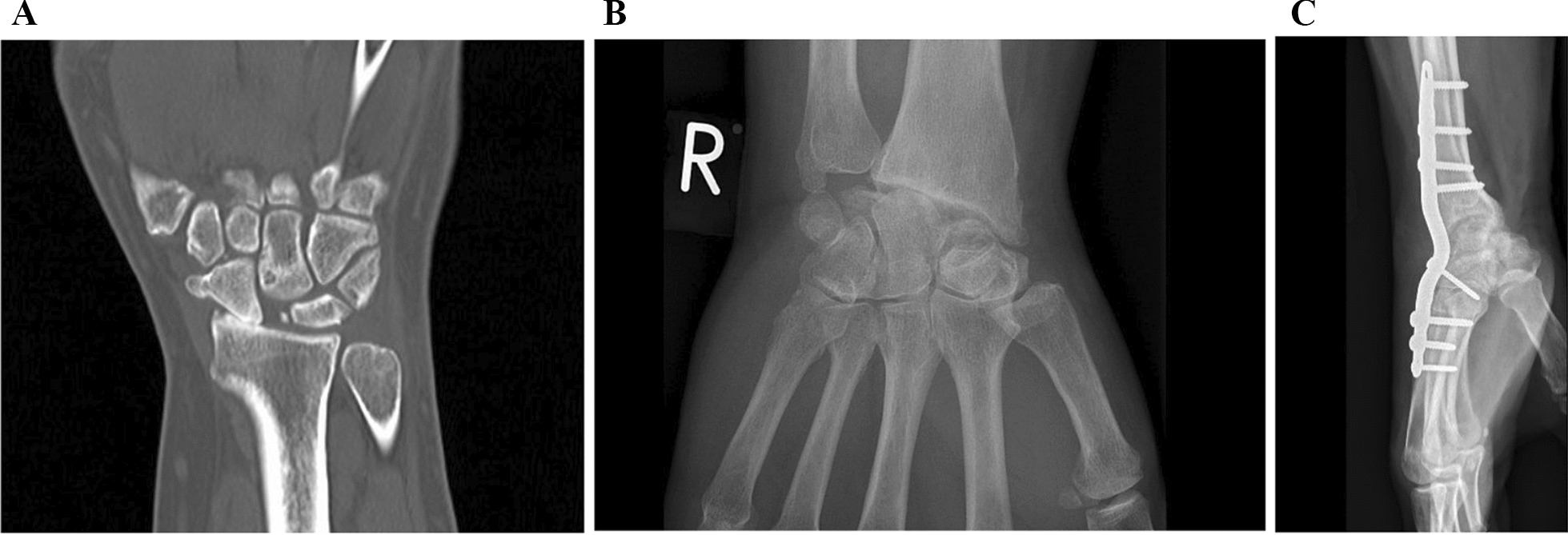
**A** 57-year-old male with a SLAC wrist. Preoperative CT scan of the right wrist (coronal plane) showing lunate fossa of the radius, free of radiological degenerative changes, justifying the PRC procedure. **B** Postoperative X-ray (P-A plan) showing clear signs of osteoarthritis, 14 months after the primary PRC procedure. **C** X-ray (side plan) of the patient’s wrist, 21 months after the primary procedure. The wrist has been fused due to severe pain

Axillary block anaesthesia is used for the procedure, with the patient in the supine position. A dorsal longitudinal skin-incision, ulnar to the Lister´s tubercle is performed. Subcutaneous nerves are preserved. The retinaculum is incised partially, between the third and fourth extensor tendon compartment. The tendons are then retracted. The posterior interosseus nerve is resected. The anterior interosseus nerve is resected through a small volar incision in the interosseous membrane. A T-shaped flap, with the transverse part proximally, is created in the dorsal wrist capsule, and the scaphoid, lunate, and triquetrum are visualized. The wrist is flexed before removing the carpals. Firstly, the scaphoid is removed, hereafter the lunate and triquetrum. It is important to preserve the volar extrinsic ligaments to stabilize the joint. After removing the carpals, the joint capsule is closed and secured by suturing the flaps to the radius, and, if necessary, using a bone anchor. The partial incision of the retinaculum is sutured with a nonabsorbable 4-0 Ethibond (Ethicon, Somerville, NJ, USA) sutures. The skin is closed with a 4-0 Ethilon (Ethicon, Somerville, NJ, USA) suture. Postoperatively, the operated wrist is immobilized in a palmar plaster of Paris for four weeks, and the patient is subsequently referred to an occupational therapist, where a removable wrist orthosis is applied for up to 2–4 weeks. 6 weeks postoperatively the patient is allowed gradually to start weight-bearing activities, and after 3 months all restrictions are lifted (Fig. 1).

#### LCF

**Fig. 2:**
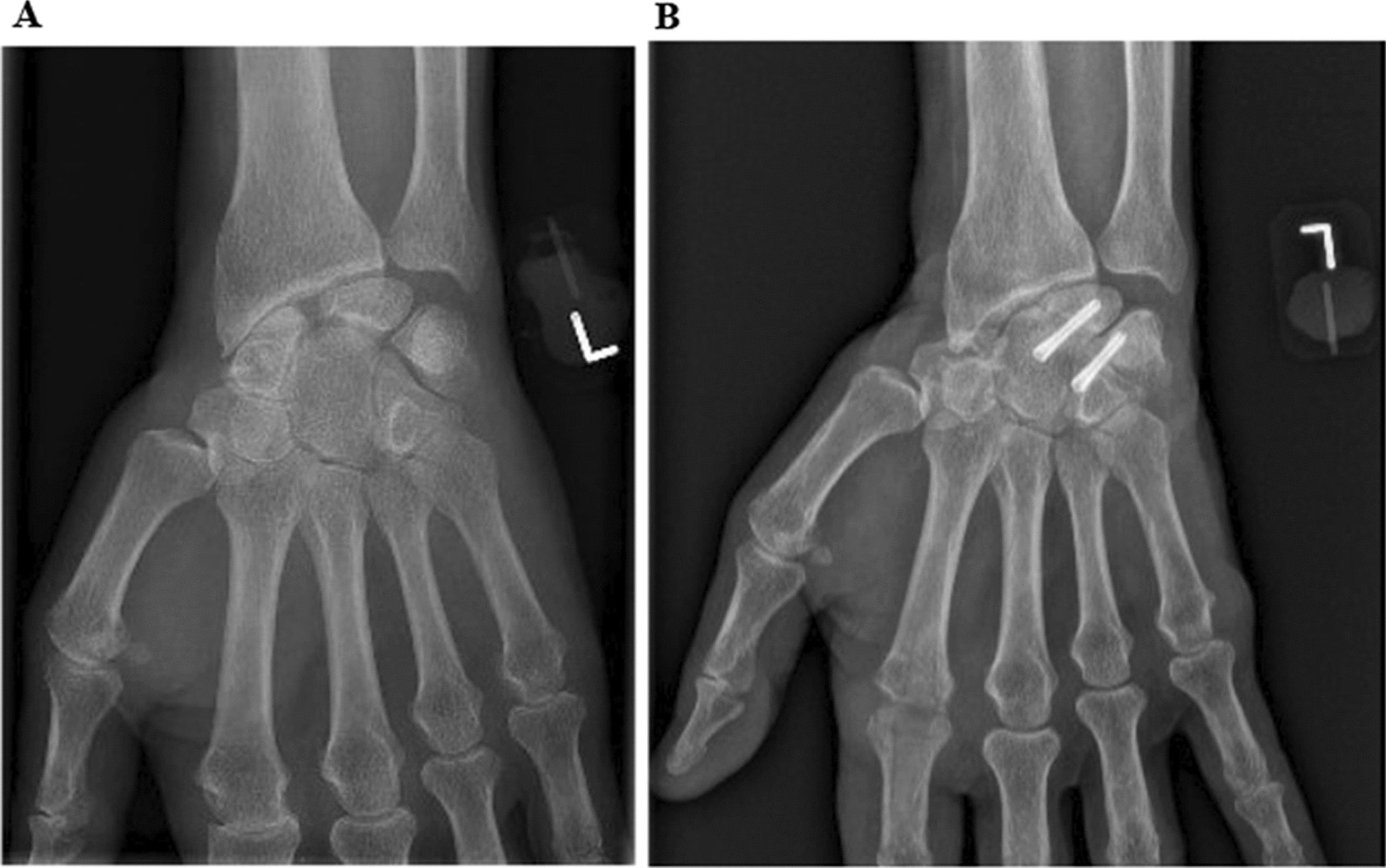
**A** 63-year-old male presenting a painful SLAC wrist. Preoperative X-rays (P-A plane) are showing signs of SLAC wrist. **B** Postoperative X-ray (P-A plane) showing no signs of osteoarthritic changes, 4 years after the 2CF surgery of the left wrist. The patient is pain-free

The patient is anaesthetized with an axillary nerve block and positioned supine. A dorsal longitudinal incision is performed with the affected hand in pronation. Sensory nerves are kept aside, and the extensor retinaculum is then opened through the 4th extensor compartment. The posterior interosseus nerve is then resected and the wrist joint capsule is opened with a radially based V-shaped flap. The scaphoid bone is identified and excised. The cartilage between the capitate and lunate bones is removed and the lunate bone is repositioned in the same axis relative to the capitate and fixed temporarily with K-wires. Final luno-capitate arthrodesis is performed with one or two Acutrak2 mini screws (Acumed, Hillsboro, Oregon, USA). The luno-triquetral and triquetro-hamate joint spaces are assessed perioperatively and arthrodesis between hamate and triquetrum is performed in the same way, if necessary. The joint spaces are left untreated if normal cartilage is identified, and if the stability of the triquetro-hamate joint could be confirmed intraoperatively, the fixation of this joint is not needed [13]. However, the presence of the lunate type 2 might lead to nonunion or postoperative pain [15]. Lunate type 2 was present in six out of 45 patients of this group and was usually treated by the removal of the triquetrum [13]. If necessary, radial styloidectomy might be added to the procedure to enhance the radial movement. Finally, the joint capsule is closed with Ethibond 4-0 sutures (Ethicon, Somerville, NJ, USA), and the skin is closed with Nylon 4-0 (Ethicon, Somerville, NJ, USA). Absorbent dressing and a dorsal plaster cast are used for 6 weeks. Suture removal and application of new dorsal plaster were performed after 2 weeks. After 6 weeks, an X-ray is performed. In case of satisfactory position and healing of the arthrodesis, the patient is referred for occupational therapy; active unloaded exercises the following 6 weeks. Subsequently, loading is allowed according to the stage of the bony union and the patient´s ability. Full weight-bearing is allowed when the healing is verified with X-rays or CT scan investigations (Fig. 2).

### Objective and subjective measurements

The primary outcome was pain in activity, and it was evaluated using the Visual Analogue Scale (VAS) ranging from 0 to 100. Zero (0) represents no pain and a hundred (100) represents the worst imaginable pain. As secondary outcomes, the following postoperative parameters were collected. Range of motion was assessed by measuring flexion, extension, radial- and ulnar deviation of the wrist, using a goniometer. The grip strength was measured on both hands with a Jamar® hydraulic hand dynamometer (North Coast Medical, Morgan Hill, USA) in kilogram-force (KgF). To measure the function and symptoms of the upper extremity, as the patient-related outcome measurement (PROM), the Quick-DASH (qDASH) questionnaire was used. The questionnaire, consisting of 11 questions, gives a value that can be calculated to a score between 0 and 100. A higher score indicates a greater disability.

### Radiological evaluation

As an additional outcome, all patients had two-plane radiographs taken at the last follow-up. The radiographs were evaluated for signs of radio-carpal osteoarthritis, postoperatively. This evaluation was performed by an independent reviewer, a hand surgeon, not involved in the given patient´s surgical treatment. The signs of osteoarthritis development were graded in five categories: none, doubtful, mild, moderate, and severe arthritis (grade 0-IV), according to Kellgren-Lawrence Classification System [16].

### Satisfaction

At the last follow-up, patients were asked by an interviewer, not involved in the surgical treatment, how satisfied they were with the surgery. The answers were divided into the following four categories: poor, fair, good, and excellent.

### Statistical analysis

The Welch Two Sample t-test was used for the postoperative continuous variables with a normal distribution, grip strength, and ROM. Whereas the Wilcoxon Rank Sum test was used for the calculations regarding non-parametric values, VAS scores, and qDASH scores. The statistical calculations were performed with the software R Studio (R Program, 2021) [17]. A* p* value less than 0.05 was considered significant (5% significance level).

## Results

The PRC group included 15 patients, 10 women and 5 men, with a mean age of 60 years (range 31–77). The mean follow-up was 42 months. The LCF group included 45 patients, 15 women and 30 men, with a mean age of 58 years (range 35–76). 33 patients underwent bi-column fusion, and 12 patients were operated on as a single-column fusion. The mean follow-up was 41 months (Table 1). The distribution of operative indications and the distribution of SLAC/SNAC stages are shown in Table 2.

**Table 2:** Distribution of operative indications

Indication	PRC	LCF
SLAC	13 (87%)	37 (82%)
SNAC	2 (13%)	8 (18%)

Stage	PRC	1/2CF
1	4 (27%)	15 (33%)
2	7 (47%)	10 (22%)
3	4 (27%)	20 (44%)

The results of the comparative statistical analysis of the measured parameters are shown in Table 3

**Table 3:** Results and comparative analysis

Variables^a^	PRC	LCF	*p *value (CI)
VAS^b^	45 (23.0–66.5)	7.5 (0.0–30.0)	0.002
Grip strength^c^	17.5 (12)	28.5 (13)	0.008 (− 18.856 to 3.019)
Men	18.2 (13)	33.7 (13)	0.03
Women	14.2 (7)	17.4 (5)	0.26
Flexion-extension^d^	57° (26)	62° (19)	0.525 (− 21.155 to 11.169)
Radial-ulnar deviation^d^	21° (15)	36° (18)	0.003 (− 26.166 to 5.504)
qDASH^e^	43 (24.5–62.5)	11 (4.8–19.5)	0.002

^a^Postoperative

^b^Pain during activity. Expressed as median (IQR)

^c^In KgF. Expressed as mean (SD)

^d^In degrees (°). Expressed as mean (SD)

^e^Quick-DASH score. Expressed as median (IQR)

*KgF* kilogram force, *SD* standard deviation, *IQR* interquartile range

### Objective and subjective results (pain, grip strength, range of motion, and qDASH)

The postoperative pain in activity was significantly lower in the LCF group, with a median of 7.5 (IQR = 0.00–30.0), compared to the PRC group, with a median of 45 (IQR = 23.0–66.5), (*p* = 0.002).

The postoperative flexion–extension was not different between the two groups (*p* = 0.525). The LCF group had a significantly better radial-ulnar deviation, with a mean of 36 degrees (SD = 18), compared to the PRC group with a mean of 21 degrees (SD = 15), (*p* = 0.003).

The LCF group had a significantly better postoperative grip strength, with a mean of 28.5 KgF (SD = 13), compared to the PRC group with a mean of 17.5 KgF (SD = 12), (*p* = 0.008).

However, when correcting for gender, grip strength was 34/18 KgF for men, and 17/14 KgF for women for the LCF/PRC groups, respectively. This difference between LCF and PRC groups was significantly better for men treated with LCF, but not different for women (*p* = 0.03, *p* = 0.27), respectively.

**Fig. 3:**
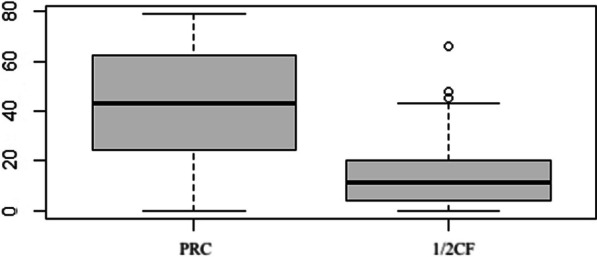
Boxplots of the postoperative qDASH scores for the two groups. qDASH = Quick version of Disabilities of the Arm, Shoulder, and the Hand questionnaire

The LCF group had a significantly lower qDASH score postoperatively, with a median of 11 compared to the PRC group with a median of 43 (*p* = 0.002). The results of the qDASH are visualized in Fig. 3.

The LCF patients had an average union time of 9.5 weeks with a range of 5–25 weeks. The union rate was 96% (43/45). Only two patients required conversion to total first fusion due to lack of healing after the primary procedure.

### Complications

It can be deduced from the data in Table 4 that 27% of the patients in the PRC group have undergone a total wrist fusion, as opposed to the LCF group where it was 19% of the patients. Out of four PRC patients needing conversion to total wrist fusion, one patient had a preoperative stage I SLAC condition, two patients had stage II, and one patient had stage III SLAC condition as an indication for the PRC surgery. The decision to perform a wrist fusion was made in symptomatic patients only, and after all the necessary pro- and contra details were taken into consideration. Concerning the other complications, it becomes apparent that there is a difference in the types of complications between the two groups, although the total complication rate in the PRC group is 60%, and 35% in the LCF group, after the follow-up of 42 and 41 months, respectively.

**Table 4:** Postoperative complications

Complications	PRC	LCF^a^
Major re-operations	4 (27%)	8 (19%)
Total wrist fusion	4	5
ReMotion TWA	0	1
Re-arthrodesis with 2CF	0	2
Minor re-operations	1 (7%)	7 (16%)
Carpal tunnel release	0	3
Removal of compression screws	0	2
AIN/PIN neurectomy	0	1
Excision of the pisiform	0	1
Other	1^b^	0
Other complications	4 (27%)	0
General edema of the hand	3	0
Postoperative infection	1^c^	0

^a^Number of patients: 43. Lost to follow-up: 2 (1 deceased, 1 psychotic)

^b^Removal of scaphoid remains

^c^Treated with peroral antibiotics with success

*TWA* total wrist arthroplasty, *2CF* two column fusion, *AIN/PIN* anterior/posterior interosseous nerve

### Postoperative osteoarthritis development

Table 5 shows a remarkably higher incidence of the development of radiological signs of osteoarthritis in the PRC group, where all the patients developed osteoarthritis of some degree at the final follow-up. Only 19% of the LCF patients developed radiological signs of osteoarthritis. The patients with these changes were not always necessarily suffering from pain.

**Table 5:** Postoperative degree of osteoarthritis

	PRC	LCF^a^
0	0	35 (81%)
I	0	4 (9%)
II	5 (33%)	2 (5%)
III	2 (13%)	2 (5%)
IV	8 (57%)	0

	PRC	LCF^b^
*Patient-reported treatment satisfaction scores*		
Excellent	3 (20%)	19 (48%)
Good	2 (13%)	14 (35%)
Fair	5 (33%)	2 (5%)
Poor	5 (33%)	5 (13%)

^a^Number of patients: 43

Lost to follow-up: 2 (1 deceased, 1 psychotic)

^b^Number of patients: 40

Lost to follow-up: 5 (1 deceased, 2 language barrier, 1 psychotic, 1 failed to reach)

### Satisfaction

83% of the LCF patients reported “Good” or “Excellent” on the satisfaction score at the last follow-up, whereas 33% of the PRC patients reported “Good” or “Excellent” (Table 5).

## Discussion

We retrospectively compared the postoperative results 3.5 years after SLAC/SNAC wrist surgery in patients treated with the PRC or LCF procedure. Our findings showed that LCF resulted in less pain in activity, better ulnar-radial flexion, and a lower Quick-DASH score, compared to the PRC group. The grip strength in men was better after LCF treatment. There was no difference between groups in the dorsal-volar range of movement and the grip strength for women. The PRC group's conversion rate to a total wrist fusion was higher. All the PRC patients had developed some degree of osteoarthritis, radiologically, whereas it was only seen in 19% of the LCF patients after the last follow-up. In addition, there was a higher satisfaction score with the procedure in the LCF group.

Previous studies have found 4CF and PRC [4, 9], and LCF and 4CF [6, 18] to be comparable. Therefore, one could hypothesize that PRC and LCF consequently would be similar. However, our study’s results favour LCF in almost all the examined outcomes.

There seems to be consensus in the literature about the volar-dorsal range of movement being best preserved after PRC compared to 4CF [4, 9, 18]. However, this differs from our results in comparing PRC to LCF, where we found no differences between groups for this measurement. The LCF group had a significantly better range of motion regarding ulnar-radial range of movement. Worth mentioning is that Saltzman et al. [9], in their systematic review between PRC and 4CF also found a greater radial deviation in the 4CF patients. The authors also reported that studies have not found a significant difference in pain and patient-reported satisfaction when looking at 4CF vs. PRC. This contrasts with our study, where there was significantly less pain and greater patient-reported satisfaction after LCF than after PRC.

In contrast to our findings, Luchetti [18] presented good clinical results after PRC, performed with a volar approach. 50 patients were reviewed, and despite X-ray investigation showing worsening in 34 patients, and severe in six, there was no correlation to the clinical function of the patients after the follow-up of 54 months. In our opinion, significantly more pain in the PRC group in our study and the simultaneous development of radiographic signs of osteoarthritis could imply that there is a correlation and high clinical relevance of these findings.

The grip strength is also generally said to be better in the 4CF patients, and therefore we also expected the LCF patients to have an equivalently better grip strength compared to the PRC patients. This was also confirmed in our findings, although these must be interpreted with caution since there was a difference in sex distribution between groups of our study giving an unintended advantage to the LCF group which contained proportionally more men than women. Although, when stratified for gender, the difference in grip strength was statistically significant for men favouring the LCF treatment method, while the difference in grip strength between treatment methods was not found for women.

Furthermore, there was far better patient-reported satisfaction in the LCF group. Our results, close to mid-term follow-up are in agreement with the study of Ali et al. [19], who also found poor patient satisfaction among PRC patients during the long-term follow-up.

As expected, compared to other studies [4, 9], we did find a higher degree of postoperative osteoarthritis in the PRC group, but we did not expect the difference between the two groups to be as apparent as found. Wall et al. [20] also found radiographic signs of osteoarthritis, in 8 out of 11 wrists of PRC patients. The authors recommend that the PRC procedure should not be performed on patients younger than 35–40 years of age. However, our PRC cohort contained a single patient under the recommended age for this treatment, a 31-year-old male with an avascular proximal pole after a chronic scaphoid nonunion. The patient underwent two surgical procedures before his PRC reconstruction: removal of the avascular tissue and the placement of an Adaptive Proximal Scaphoid Implant (APSI), and repositioning of the APSI implant after the luxation episode, one year and 6 months, respectively, before final APSI implant removal and PRC procedure. The patient recovered fully and did not experience either pain or need for further surgery, although the final radiologic x-ray showed some degree of osteoarthritis.

Concerning the complications, Saltzman et al. [9] reported general oedema of the hand as a complication in the PRC group, while decompression of the median nerve was a complication in the 4CF group. This was a distribution also found in our study (Table 4). However, it should be brought to attention, that in the present study, the PRC group had more conversions to total wrist fusion compared to the LCF group. Furthermore, the total complication rate was substantially higher for the PRC group than for the LCF group (Table 4). The conversion rate in the LCF group is supported by the findings in the study by Ozyurekoglu et al. [21], where there is an even lower conversion rate (3%) in the 4CF cohort of 32 patients using the same fixation device as our study, however, reported after the short term follow-up of average 8 months (range 6–64 months).

Preoperatively, the LCF group had more patients with SLAC/SNAC stage 3 (Table 2). It should be noted that it is not recommended to perform the PRC procedure if the patient has SLAC/SNAC above stage 2 [18]. Surgeons´ preference towards using the PRC technique in these patients could be based on various reasons and this might have contributed to the worse result in the PRC group. In our study, four PRC patients had SLAC/SNAC stage 3 preoperatively, as shown in Table 2. However, of these, only one ended in conversion to total wrist fusion.

Nevertheless, some of the arguments for using PRC vs. 4CF are that the PRC procedure is a simple and less invasive procedure, with a shorter time to recovery and fewer complications [4, 9, 17]. The same arguments do not hold up compared to the LCF, as these procedures are technically simpler to perform, needing less hardware and using a joint capsule-sparing approach. Thus, the LCF surgical methods are less invasive than the 4CF, have fewer complications, resulting in a high union rate and better mobility [6, 13, 21, 22]. Even though the LCF is a simpler procedure than the 4CF, the PRC is the simplest surgical procedure of these. Therefore, the experience of the surgeon and the functional level and demands of the patients should be taken into consideration when planning the surgery.

Our study has the following limitations: first, our study is not randomized, and there is a risk of selection bias in the groups that could affect the postoperative outcomes. Second, there is a different distribution of sex in the two groups, which most likely affected the grip strength outcomes. This was found favourable after the LCF treatment in men but not in women, when patients were stratified for sex. Finally, we do not compare preoperative data, as those were lacking for some parameters in the PRC group. Nevertheless, previous studies [4] have already looked at preoperative vs. postoperative outcomes, of PRC and 4CF, and found them comparable. Besides, in our study, the preoperative stages of the degenerative changes between both treatment groups were similar and slightly worse in the LCF group.

Despite that 4 out of 15 patients from the PRC group had preoperatively stage 3 of SLAC condition, a non-recommendable stage, the overall results of this retrospective study clearly point towards PRC being inferior compared with LCF, although the results of LCF itself may show similar deterioration pattern on the longer postoperative timeframe.

In conclusion, after 3.5 years, most findings favour LCF compared to PRC, when retrospectively comparing the two different surgical methods for a similar patient cohort of patients treated for SLAC and SNAC wrist conditions. Further, preferably prospective, or randomised, and long-term studies are needed to support or oppose our findings.

## Data Availability

All the data and materials of this study are available at the inquiry to the corresponding author.

## References

[1] Watson HK, Ryu J (1986). Evolution of arthritis of the wrist. Clin Orthop Relat Res.

[2] Kirschenbaum D, Schneider LH, Kirkpatrick WH, Adams DC, Cody RP (1993). Scaphoid excision and capitolunate arthrodesis for radioscaphoid arthritis. J Hand Surg Am.

[3] Shah CM, Stern PJ (2013). Scapholunate advanced collapse (SLAC) and scaphoid nonunion advanced collapse (SNAC) wrist arthritis. Curr Rev Musculoskelet Med.

[4] Mulford JS, Ceulemans LJ, Nam D, Axelrod TS (2009). Proximal row carpectomy vs four corner fusion for scapholunate (Slac) or scaphoid nonunion advanced collapse (Snac) wrists: a systematic review of outcomes. J Hand Surg Eur.

[5] Strauch RJ (2011). Scapholunate advanced collapse and scaphoid nonunion advanced collapse arthritis–update on evaluation and treatment. J Hand Surg Am.

[6] Gaston RG, Greenberg JA, Baltera RM, Mih A, Hastings H (2009). Clinical outcomes of scaphoid and triquetral excision with capitolunate arthrodesis versus scaphoid excision and four-corner arthrodesis. J Hand Surg Am.

[7] Aita MA, Nakano EK, Schaffhausser HL, Fukushima WY, Fujiki EN (2016). Randomized clinical trial between proximal row carpectomy and the four-corner fusion for patients with stage II SNAC. Rev Bras Ortop.

[8] Amer KM, Thomson JE, Vosbikian MM, Ahmed I (2020). Four-corner arthrodesis versus proximal row carpectomy for scapholunate advanced collapse: a systematic literature review and meta-analysis. Ann Plast Surg.

[9] Saltzman BM, Frank JM, Slikker W, Fernandez JJ, Cohen MS, Wysocki RW (2015). Clinical outcomes of proximal row carpectomy versus four-corner arthrodesis for post-traumatic wrist arthropathy: a systematic review. J Hand Surg Eur.

[10] Singh HP, Dias JJ, Phadnis J, Bain G (2015). Comparison of the clinical and functional outcomes following 3- and 4-corner fusions. J Hand Surg Am.

[11] Van Nuffel M, Vanhees M, Maeckelbergh L, Degreef I, De Smet L (2020). Four-corner fusion versus proximal row carpectomy : a retrospective review with a minimal follow-up of 9 years. Acta Orthop Belg.

[12] Imbriglia JE, Broudy AS, Hagberg WC, McKernan D (1990). Proximal row carpectomy: clinical evaluation. J Hand Surg Am.

[13] Solgaard L, Gvozdenovic R (2023). Single- and bicolumn Limited Intercarpal Fusion: a solution for the SLAC or SNAC wrist. J Wrist Surg.

[14] Tang JB, Giddins G (2016). Why and how to report surgeons’ levels of expertise. J Hand Surg Eur.

[15] Gauci MO, Waitzenegger T, Chammas P-E, Coulet B, Lazerges C, Chammas M (2020). Comparison of clinical outcomes of three-corner arthrodesis and bicolumnar arthrodesis for advanced wrist osteoarthritis. J Hand Surg Eur.

[16] Kellgren JH, Lawrence JS (1957). Radiological assessment of ostheoarthrosis. Ann Rheum Dis.

[17] R Core Team (2021). R: a language and environment for statistical computing.

[18] Luchetti R (2018). Proximal row carpectomy, scaphoidectomy with midcarpal arthrodesis or midcarpal tenodesis: when and how to use. J Hand Surg Eur.

[19] Ali MH, Rizzo M, Shin AY, Moran SL (2012). Long-term outcomes of proximal row carpectomy: a minimum of 15-year follow-up. Hand (N Y).

[20] Wall LB, Didonna ML, Kiefhaber TR, Stern PJ (2013). Proximal row carpectomy: minimum 20-year follow-up. J Hand Surg Am.

[21] Ozyurekoglu T, Turker T (2012). Results of a method of 4-corner arthrodesis using headless compression screws. J Hand Surg Am.

[22] Abdelaziz AM, Aldahshan W, Elsherief FAH, Wahd Y, Soliman HAG, El Behairy HF (2020). Scaphoid excision with lunatocapitate fusion for the treatment of scaphoid nonunion with advanced collapsed wrist. Int Orthop.

